# Mechanics of the Prey Capture Technique of the South African Grassland Bolas Spider, *Cladomelea akermani*

**DOI:** 10.3390/insects13121118

**Published:** 2022-12-05

**Authors:** Candido Diaz, John Roff

**Affiliations:** 1 Biology Department, Vassar College, Poughkeepsie, NY 12604, USA; 2 Independent Researcher, Pietermaritzburg 3201, Kwazulu-Natal, South Africa

**Keywords:** Cyrtarachninae, kinematics, arachnology, behavior, spin, environments

## Abstract

**Simple Summary:**

Spiders are an order of organisms with highly diverse predatory techniques. All species produce silk and utilize varying degrees of adhesion to ensnare and trap prey long enough to envenomate them. Many spider families can be distinguished by their prey capture strategies, the silk structures they create, and the mechanical properties of the silk they spin. Understanding the diversity and function of these glues has much to teach us about natural bioadhesives and has application to our own synthetic adhesives. The most derived orb-webs are spun by bolas spiders, consisting of only a single capture thread, lined with a few glue droplets—often only one at the end. This web reduction must be accompanied by a strong glue. Additionally, the species *Cladomelea akermani* consistently spins its bolas and bounces. We use high-speed video to observe the prey-capture technique of *C. akermani*. The spider’s willingness to spin allowed us to record and measure the kinematics of their unique bouncing, bolas spinning behavior, and overall prey capture technique. We then tested the additional hypothesis that this bouncing behavior serves an additional purpose in pheromone distribution by creating a computational fluid dynamics model. Spinning in an open environment creates turbulent air, spreading pheromones further and creating a pocket of pheromones. Conversely, spinning within a tree does little to affect the natural airflow.

**Abstract:**

Spiders use various combinations of silks, adhesives, and behaviors to ensnare prey. One common but difficult-to-catch prey is moths. They easily escape typical orb-webs because their bodies are covered in tiny sacrificial scales that flake off when in contact with the web’s adhesives. This defense is defeated by spiders of the sub-family of Cyrtarachninae—moth-catching specialists who combine changes in orb-web structure, predatory behavior, and chemistry of the aggregate glue placed in those webs. The most extreme changes in web structure are shown by the bolas spiders which create only one or two glue droplets at the end of a single thread. They prey on male moths by releasing pheromones to draw them close. Here, we confirm the hypothesis that the spinning behavior of the spider is directly used to spin its glue droplets using a high-speed video camera to observe the captured behavior of the bolas spider *Cladomelea akermani* as it actively spins its body and bolas. We use the kinematics of the spider and bolas to begin to quantify and model the physical and mechanical properties of the bolas during prey capture. We then examine why this species chooses to spin its body, an energetically costly behavior, during prey capture. We test the hypothesis that spinning helps to spread pheromones by creating a computational fluid dynamics model of airflow within an open field and comparing it to that of airflow within a tree, a common environment for bolas spiders that do not spin. Spinning in an open environment creates turbulent air, spreading pheromones further and creating a pocket of pheromones. Conversely, spinning within a tree does little to affect the natural airflow.

## 1. Introduction

Spiders are an order of organisms with highly diverse predatory techniques [1,2]. All species produce silk and utilize varying degrees of adhesion to ensnare and trap prey long enough to envenomate them [1]. Many spider families can be distinguished by their prey capture strategies, the silk structures they create, and the mechanical properties of the silk they spin [1,2]. Orb-weavers are largely a generalist group, known for their wagon wheel-shaped webs, which primarily focus on the capture of prey mid-flight [1,2]. The most derived spiders can produce upwards of seven different silks with unique mechanical properties; five of which are used in the construction of their webs [1,2].

As generalists, orb-weaving spiders typically eat any insect that is stuck in their web. One common but difficult-to-catch prey are moths. While plentiful in most ecosystems, the powder which covers their bodies consists of sacrificial scales which flake off when in contact with a spider’s web [3,4]. As the moth thrashes, it releases them from the web, escaping. This niche has led to the evolution of a subfamily of spiders that have all evolved to specialize in moths only. The subfamily, Cyrtarachninae, consists of spiders that create various web structures sometimes limited to specific microenvironments [4,5,6,7,8]. Each of these species has adjusted their webs, behavior, and glue to overcome the superhydrophobic nature of a moth’s body while reducing the overall structure and reliance on capture thread production [7,8,9,10,11]. Such examples consist of the genera *Cyrtarachne* and *Paraplectana* whose webs are horizontal with long dangling threads and *Pasilobus* whose webs are small, triangular, and only consist of three to four strands [6,7,9,11].

The most derived orb-webs are spun by bolas spiders, consisting of a single capture thread, lined with a few glue droplets—often only one at the end [12,13,14,15,16]. Bolas spiders consist of several genera all belonging to Cyrtarachninae [14,17]. They prey on male moths by releasing pheromones to draw them close, mimicking a female; they can even change the species of moth they are hunting throughout the evening [12,13,15,16]. From here, prey capture techniques seem to vary even between spiders using the same “weapons”, such as the bolas wielders [18,19,20,21]. For instance, when a moth approaches the American species, *Mastophora hutchinsoni,* which holds a bolas consisting of one glue droplet very close to itself, it flicks the droplet at prey snagging it in a single strike [13,16,18,19]. These short-range bolas spiders respond only to the sound of their prey’s wingbeats [22]. Other species are more active and less discerning. Species of the Genus’ *Ordgarius* and *Exechocentrus* construct longer bolas of up to four droplets. Their attack behavior is easier to elicit, responding to the sound of human singing or a passing car, twirling their bolas in a circle [17,23,24,25]. The most active species, and the topic of this paper, belong to the genus *Cladomelea*. The South African grassland bolas spider species *Cladomelea akermani* constructs a bolas of up to three droplets and rotates its glue droplets and body during prey capture by spinning in a circular fashion. This behavior starts at sundown with or without prey [20,21,26,27]. While the unique behavior of many bolas spider prey capture systems has been observed in an ecological context, the exact kinematics of most bolas prey capture techniques have yet to be fully elucidated [13,15,16].

We are interested in the variation of the kinematics involved in prey capture, and how the behavior and mechanical properties of the glues utilized by moth-specialist spider species vary alongside it. In specific, we are interested in how prey capture techniques vary within bolas spiders species that construct varying bolas structures. Here, we use high-speed video to observe the kinematics of the prey-capture technique of *C. akermani*. Though we were unable to record a successful prey capture event, the spider’s willingness to spin allowed us to record and measure the kinematics of their unique self-spinning/bouncing, bolas spinning behavior, and overall prey capture technique. We aim to understand the physical and mechanical differences in prey capture between this species and *M. hutchinsoni*, their non-spinning bolas-wielding American cousins. Firstly, we aim to verify the assumption that the spinning of the spider is directly related to the spinning of its bolas by measuring the speed, period, and rotational phases of the two. We then examine why *C. akermani* chooses to bounce during prey capture, an energetically costly behavior. Our hypothesis is that while the bouncing behavior may help to spin the glue droplet, it is also helpful to spread pheromones, widening its effective hunting area. We test this by creating a computational fluid dynamics model of airflow with and without a spinning spider within an open field and compared it to that of airflow in a tree environment, like that of *M. hutchinsoni* [15,16,18,19].

## 2. Materials and Methods

### 2.1. Field Observations and Kinematics of Prey Capture

Two adult female specimens of *C. akermani* were observed over three nights at Cumberland Nature Reserve (−29.513428, 30.505196, permit OP 2233/2022). Observations began at sundown as they transitioned from resting, to actively questing (waving their front legs in the air, Supplementary Video S1), to creating a bolas and spinning it. Once the spider had begun to make a bolas, a single Baslar acA1300–60gmNIR ACE camera was set up perpendicular to the horizontal plane of the spider; distances between the camera and the spider varied depending on the position of the spider relative to surrounding vegetation. Prey capture events were filmed at 83.6 frames per second (fps), the highest speed for the resolution of our camera, using a Fujinon 12.5 mm 2/3” lens. Since most insects and arachnids do not rely on the red light for vision, subjects were illuminated using an ABI LED 54W near-infrared light (880 nm) to provide adequate lighting without impacting the behavior of the spider or moths [1].

**Figure 1:**
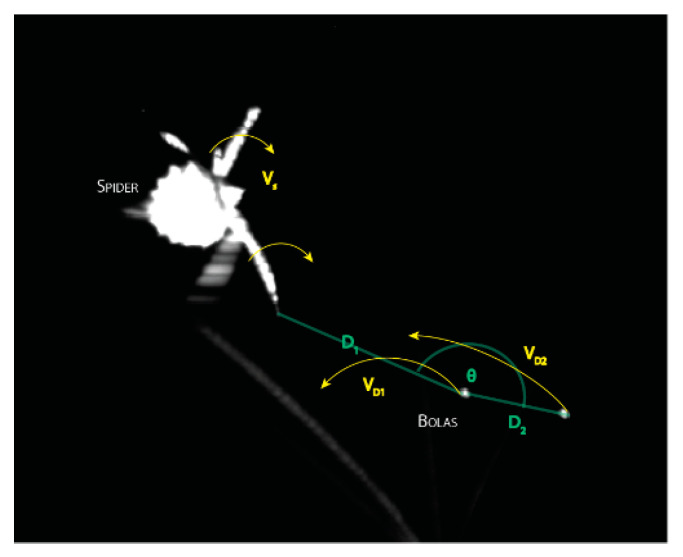
Tracking of Prey Capture Technique. Videos were tracked using Kinovea. Up to four locations were manually digitized for the position which was used to calculate the rotational velocity (*V*), period, and rotational diameter. The angle formed by the glue droplets was calculated over the course of the video using the law of cosines and the measured distances. The coordinate system is located below the spider perpendicular to the anchoring leg of the spider.

*C. akermani* performs its capture technique without the direct presence of prey [21,27]. This allowed us to observe the creation and spinning of many bolas (N = 7) over only a few nights. From the videos, the movements of the spider and glue droplet were manually tracked using the open-source kinematics software Kinovea [28] (Figure 1). Up to four positions were tracked: (SA) the tip of the leg the spider uses to spin itself, (AA) the anchoring leg the spider holds the bolas with, and up to two glue droplets (D1, D2) located on the bolas. Each position was tracked during the entirety of each video which varied between 4 and 20 rotations in length. The position was tracked as a radial vector from a coordinate system located below the spider, perpendicular to the center of the spider. Rotational velocities were calculated using the finite difference in position and the known time interval between frames, measured as peak displacement using the measured diameter of rotation for that particular revolution. For videos in which the bolas were made of two droplets, we also measured the angle formed by the middle droplet, the end of the bolas, and the spider’s anchoring leg using the law of cosines (Figure 1). For all values, averages and standard errors were calculated. A mixed ANOVA was run to determine if the four tracked locations varied statistically in their rotational period, velocity, and diameter. Radial distances were plotted and used to find the phase difference between the spider’s legs and glue droplet maximum displacement.

### 2.2. Pheromone Airflow—Computational Fluid Dynamics Model

When sundown begins, *C. akermani* sets up its bolas and begins to spin, continuing to do so with or without the apparent presence of prey. This raised a question of whether or not this bouncing behavior serves a purpose other than to spin their bolas, as this is a timely and energetically costly behavior that is not shared with every other bolas species [16,17,27].

**Figure 2:**
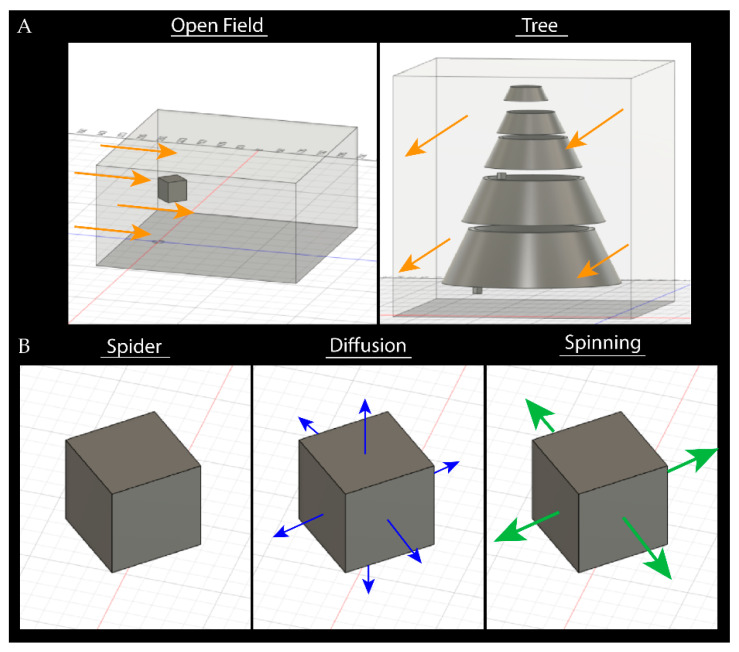
Environment models and flow conditions used in computational fluid dynamics modeling. (**A**) Open field tests involved a rectangular environment open on all sides except the bottom. Orange arrows show the direction of the wind, behind the spider, at 3 m/s. The tree model was made of five rings, and two spiders were placed due to being a larger structure, one at the top and one at boom. (**B**) The spider and pheromone cloud were modeled as a perfect cube, with sides of length 1 cm. The diffusion condition applied a small velocity, 0.05 m/s, exiting all faces of the cube. The spinning condition applied a larger velocity, 3 m/s, exiting the four front and back faces of the spider.

We made simple models of the environments of *C. akermani* and *M. hutchinsoni*, an open grassland, and a large arboreal structure respectively, using Autodesk Fusion 360 (Figure 2A) [29]. These models were then imported as .step files into Autodesk CFD 2023 where four varying flow condition tests were performed: (1) a small breeze at 3 m/s, (2) slow diffusion at 0.05 m/s, (3) the magnus effect of a spinning spider at 3 m/s, and (4) the combination of a spinning spider and a breeze (details listed below) [30].

In each model, the spider was represented as a cube-shaped volume of pheromones, using the default material properties of ‘Air’ material (Figure 2B). The field model consisted of a large open rectangular area with only boundary conditions on the floor. Within the field, results were shown as the airflow velocity along three planes parallel to the wind: one in the plane of the spider, one slight above, and one slightly below. Our tree model is composed of four increasingly smaller plates, forming a cone shape, to mimic the open space of the leaves where the spider hangs. For the tree conditions, two spiders were placed within the confines of the tree with one at the bottom and one at the top (Figure 2A). The flow is shown by four planes (1) bisecting the tree, parallel to the tree trunk, (2) perpendicular to the wind flow, just before the wind contacts the tree, (3) a plane parallel to the flow of wind and located directly below the opening of the canopy, and (4) a plane parallel to the wind bisecting the tree halfway up.

**Condition** **1.** **Wind**—We model air flow within the field as laminar air flow through a wind tunnel open on all sides, originating behind the spider (within the field) and/or tree, 3 m/s (Figure 2A).

**Condition** **2.** **Diffusion**—We model the diffusion as a very low distribution over time in all directions, 0.05 m/s (Figure 2B). Within our models, this diffusion was modeled as originating out of the spider in all directions, as 6 sides of a cube. For this behavior, we expect the flow to be extremely low and slow. Diffusion can be described by Fick’s first law which relates the diffusive flux to the gradient of the concentration, in that a solute will move from a region of high concentration to a region of low concentration across a concentration gradient [31]. These low forces cause diffusion to be a relatively slow method of distributing pheromones. Equation (1) shows the general form of diffusion flux, *J*, the amount of substance per unit area per unit time. *D* is the diffusion coefficient, *φ* is the concentration, and *x* is the position.

(1) $$J=-D\frac{d\varphi }{dx}$$

**Condition** **3.** **Bouncing**—As *C. akermani* bounces its large legs, it creates areas of low pressure which forces the pheromones around to move away from the spider. This effect, similar to a rotating baseball, except that now occurs in both directions as the spider alternates, is known as the Magnus effect [32]. The air flow speed is shown by Equation (2) where *s* is the rotation rate (revolutions per second), *ω* is the angular velocity of spin (radians/second) and *r* is the radius of the cylinder (meters). This means the resulting magnus effect is proportional to the spinning speed and the airflow behind them.

(2) $$G={\left(2\pi r\right)}^{2}*s=2\pi r^{2}\omega$$

This equation shows that the airflow created by the spider should be outwards from its motion, like that coming off a hand fan. To model this cyclical bouncing, we used air flow velocity pointing outward and parallel to the ground. We model this as vectors flowing outward of the four faces in the plane of airflow. This excludes the faces pointing to *y*-axis. As the spider moves, it creates a wafting force in all directions, half within the direction of the wind and half against it. Because it is bouncing and not spinning the force alternates left and right, fanning the pheromones, instead of consistently pushing it in one direction, 3 m/s (Figure 2B).

**Condition** **4.** **Bouncing and Wind**—This condition is the most equivalent to that of the *C. akermani* and utilizes both wind and bouncing conditions listed above at the same time.

## 3. Results

### 3.1. Observations on Bolas Building Behavior and Kinematics of Prey Capture

**Figure 3:**
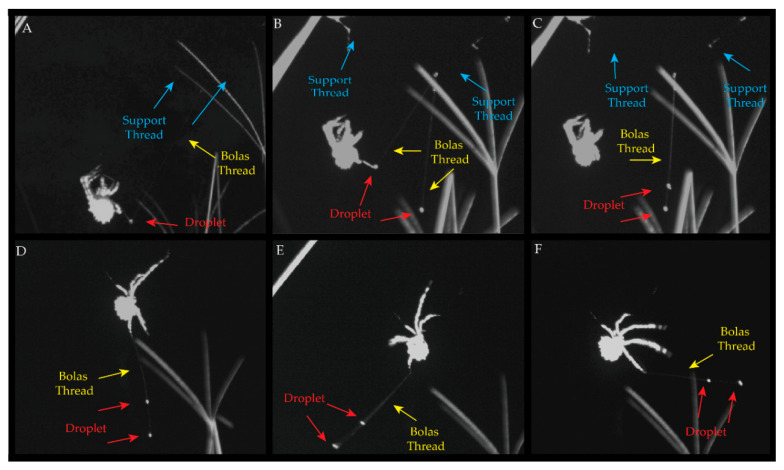
Breakdown of Multi-Bolas Creation and Prey Capture Behavior. The spider shown has a carapace width of 12 mm. Red arrows in each image are glue droplets and yellow arrows show the bolas capture thread. *C. akermani* sits on its grass or leaf until sundown. Like other spiders it first moves up and along the top of its leaf or grass stalk, creating a simple beam. It then waves its long legs in the air, wafting them about. (**A**) the spider creates two support threads (blue arrows), sliding down one and attaching a bolas to one strand. It lets the droplet slowly fall away as it gets larger. (**B**,**C**) then moves back up to the top anchoring another thread to the support beam. It drops back down again, but not as far as previous, and creates another glue droplet, which is allowed to swing into the other strand, making a single overall structure. (**D**) Once finished, the spider orients itself parallel to the support bar, grabs the bolas with its lower second leg, and holds its especially long and hairy front legs out. (**E**) It spins the bolas in a circle with its anchoring second leg. (**F**) after it has a consistent and circular motion started with the droplets, the spider begins to swing its front legs back and forth, spinning/bouncing.

*Cladomelea akermani* sits on a grass stalk or leaf until sundown when it begins to be active, waving its long legs into the air before moving from its resting spot. It starts creating a bolas by first moving up and along the top of its leaf or grass stalk and creating a simple beam. It then reinforces the beam, moving across it, as all other spiders do during web preparation [1]. As it does this, it waves its long legs in the air, wafting them about (Supplementary Video S1). This could be for multiple reasons, which are untested, including sensing for moth activity on the breeze, testing the wind speed, and/or distribution of pheromones. After the beam is reinforced *C. akermani* moves towards one side of the beam, releasing silk. At a distance of ~5–6 cm, the spider lowers itself, extruding another thread. There it dangles its silk, now connected to two threads, creating a V-shape. Dropping a bit further the spider begins to attach a bolas to the strand it is not dangling from. Pulling out large amounts of flagelliform and aggregate glue, the spider pushes it together into a large ball, letting the bolas slowly fall away as it gets larger, ultimately swinging away (Figure 3A, Supplementary Video S2). When the spider decides to make a bolas with multiple glue droplets, it then moves back up to the top anchoring an additional thread and making another v-formation (Figure 3B, Supplementary Video S3). It lowers itself again but not as far as previously. It repeats its glue droplet-making process and the droplet is allowed to swing into the previously constructed strand, forming a single overall structure (Figure 3C).

Once finished, the spider orients itself parallel to the support bar, grabs the bolas with its lower second leg, and holds its especially long and hairy front legs out (Figure 3D). It spins the bolas in a circle with its anchoring leg, second leg (Figure 3E). After it has a consistent and circular motion started with the droplets, the spider begins to swing its front legs back and forth, bouncing on the line, twisting but never fully rotating (Figure 3F, Supplementary Videos S4 and S5).

We were not able to record a moth actively being caught with our camera during this trip, but we did observe moths’ approach. They arrived rapidly from several meters downwind of the spider and flew in an indirect almost spiral fashion, towards the spider. Though close the spinning bolas failed to strike the moth, which then flew away. Throughout the evenings, very few moths closely approached the spider, though there was observable moth activity in the area immediately surrounding the spider all night. Moths were observed doing numerous things, such as sitting in the grass directly below the spider and surrounding it. Some moths climbed the grass stalks the spider was anchored on, moving up and down them, while fluttering their wings. However, those moths never approached the spider or flew near it.

**Figure 4:**
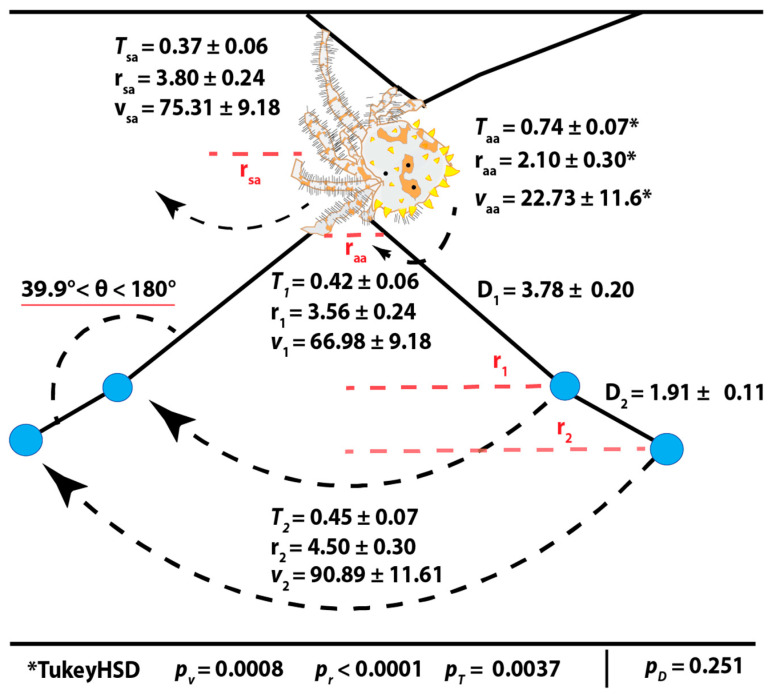
Average Kinematics of Spider Legs and Bolas Glue Droplets During Spinning. *Post hoc* Tukey analysis showed that the spinning of the anchor leg is the most statistically different for all measured values (period *T* in s, velocity *v* in cm/s, and radius *r* in cm). The anchor leg barely moves and completes only half a rotation for each one of the glue droplets that the swing leg completes. Though the large variation in spinning speed led to no overall difference within tests, the D_2_ was always spinning faster and further out than D_1_. The angle between D_1_ and D_2_ can be seen changing while spinning leading to variation in the angle between them (Supplementary Data File S1, Excel Datasheet).

Throughout our seven recorded videos, all measured values (period *T*, velocity *v*, and radius *r*) were highly variable for both the spider and bolas glue droplets (Figure 4). Post hoc Tukey analysis showed the anchoring leg (AA) was always statistically lower in all values than that of the swing leg (SA) of the spider and the glue droplets. Within each video, the maximum rotation of D_2_ was always greater than D_1_, 1.28 ± 0.13 times. The velocity similarly was always 1.37 ± 0.11 times higher, while the period was the same at 1 ± 0.21 s. D_2_ had the overall largest values with a maximum rotational diameter of 11.52 cm, and spinning upwards of 150.09 cm/s. The angle formed by D_1_, D_2_, and AA showed a variation between the 180° of a straight line and 39.9°.

**Figure 5:**
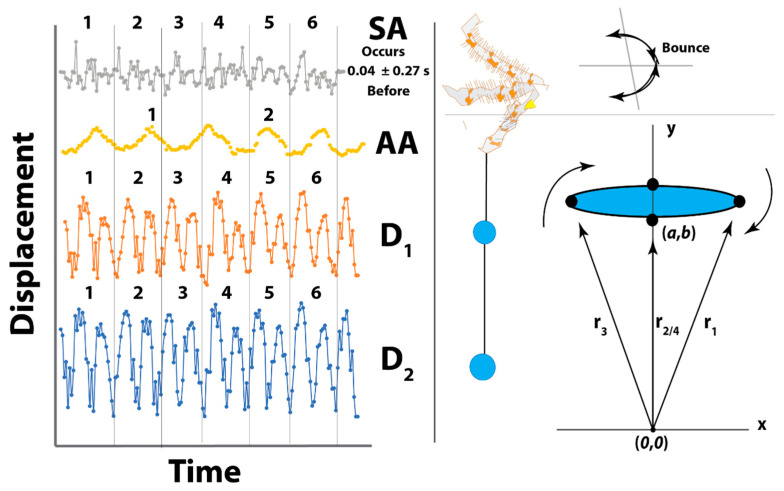
Leg and Glue Droplet Rotational Phase Alignment. Because all distances were measured as vectors radiating from a common origin, rotational periods are shown as two maxima, as the maximum displacement occurs twice. The second rotation or the second half of the circle is always shorter and slower, meaning that the energy the spider is putting into the thread is falling off almost instantly. Thus, two peaks show the period, and the rotational diameter is the maximum displacement of both peaks combined. Calculating the frames between distances, we found that the displacement of droplet two (D_2_) was always higher than droplet one (D_1_) but D_1_, D_2_, and the swing leg (SA) were nearly perfectly in sync. The maximum displacement of the SA always occurred two frames or 0.04 s before the droplets, meaning it was using its body to accelerate the glue droplets. The SA does not have two peaks because the spider is not completing a full rotation, but instead is thrusting forward and bouncing back. The period of the anchor leg (AA) was always twice as long as the other periods.

The swing leg bounces only after a firm rotation of the glue droplets has been achieved by slowly spinning the anchor leg. Then the spider begins to throw its body, increasing glue droplet rotational diameter and speed, confirming the swing leg is aiding but not necessary to rotate. Once the glue droplets are in motion and the spider begins to swing its body. This syncs the rotational period for the droplets and swing leg (Figure 5). This means the spider is thrusting its body forward and then bouncing back in the time it takes for a single rotation of the glue droplets. Calculating the phase lag between maximum displacements showed the highest displacement of the swing leg always occurred one or two frames before the maximum of each glue droplet. The period of the anchor leg was twice of others, meaning it is not fully in sync and not the driving force behind of rotation during swinging (Figure 5).

### 3.2. Pheromone Airflow—Computational Fluid Dynamics Model

**Figure 6:**
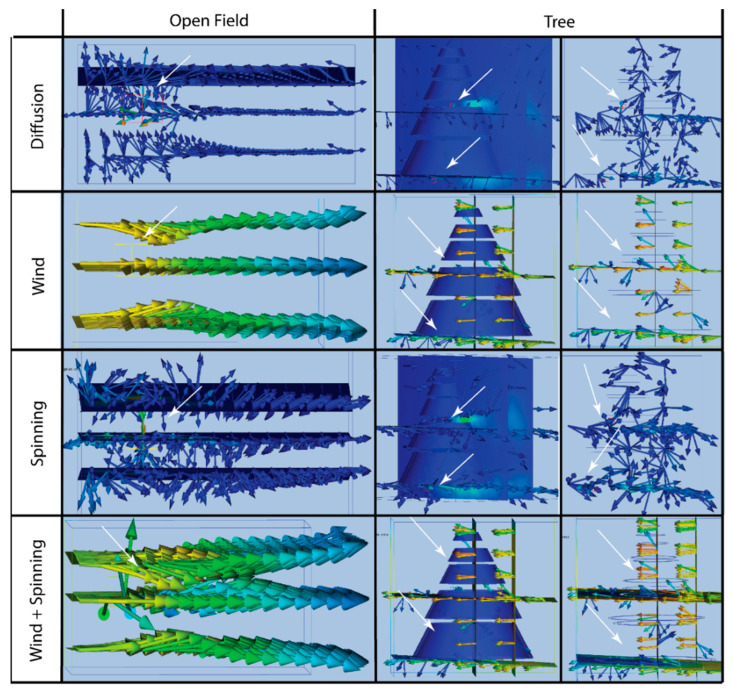
Pheromone Airflow—velocity field results of open field and tree conditions. Each image shows the Autodesk CFD velocity vector results. Air velocities are coded by a color gradient where blue is slower, and red is faster (colors are relative within each test). White arrows show the location of the spider in each test, one in the field and two in the tree. The tree tests are shown with the full tree structure and with only an outline of the tree to view airflow within the tree more easily. Open field conditions—The open field shows how diffusion creates minimal velocity around the spider. The spinning creates a larger velocity but mostly creates turbulence around the spider which continues further downstream. The wind has the largest effect on airflow. It slows down slightly as it passes through the nearly stagnant pheromone cloud, but the stream remains laminar and independent. When spinning is added to the wind, the velocity in front of the spider is increased. The normally straight trail of pheromones is instead mixed up and down being carried by the wind in now multiple directions, further distributing the pheromones over the open landscape. Tree conditions—The various barriers and openings within the tree innately create pockets of higher and lower-density air as the wind blows past it. The wind is pulled through the tree, swirling around, before being pulled out the opposite side. The highest flow velocities are surrounding the tree and exiting the tree downwind. The structure of a tree allows pheromones to be distributed throughout a tree more easily and naturally. Pheromones fill the tree and then leak from it, creating a beacon. The velocities within the tree fall when the spider bounces, slowing down the air flow in the tree.

Diffusion creates minimal velocity around the spider, most easily visualized in open field conditions (Figure 6). The spider’s bouncing and spinning creates a larger velocity in front, and a lower velocity behind it, creating turbulence. This forces pheromones to distribute further above, below, and in front of the spider. The largest airflow was created by the wind in both test environments. In the field, the wind will slow down slightly as it passes through the nearly stagnant pheromone cloud, but the streams remain laminar and independent (Figure 6). When spinning is added to the wind, the velocity in front of the spider is increased from 300 cm/s to 350 cm/s. The normally straight pheromone trail becomes turbulent, as air downwind becomes mixed both above and below the plane of the spider. These trails are further carried downwind, now in multiple directions. The spinning additionally forces pheromones behind the spider to collide with the oncoming wind. This forces air upward behind the spider at 400 cm/s. This would further distribute the pheromones over the open landscape. The various barriers and openings within the tree innately create pockets of higher, 500 cm/s, and lower-density air as the wind blows past it (Figure 6). The wind is pulled through the tree, swirling around, before being pulled out the opposite side. The highest flow velocities are surrounding the tree and exiting the tree downwind. The velocities within the tree fall when the spider bounces, slowing down the air flow in the tree to 250 cm/s.

## 4. Discussion

To our knowledge, we perform here the first kinematic descriptions of the spinning behavior of the bolas spider *Cladomelea akermani*. We used a high-speed video camera to observe their prey capture technique and track the spider’s legs as it actively bounced its body and spun its bolas. Through this, we confirmed the previously stated hypothesis that the bouncing behavior of the spider helps to increase the spinning speed and rotational diameter of the bolas; observed as the largest displacement of the swing legs always occurring before the droplet’s highest velocity and displacement. We then tested the additional hypothesis that this spinning behavior, an energetically costly one, serves an additional purpose in pheromone distribution; we tested this by creating a computational fluid dynamics model of airflow within an open field and compared it to that of airflow within a tree, a common environment for bolas spiders that do not spin.

Our question was, what is advantageous about the spinning behavior of *C. akermani* and how does it correlate with their hunting environment? Why is this additional energy expenditure worth it? To answer this, we looked at the flow of pheromones in their respective environments. We believe differences in behavior correlated with two things (1) environment and (2) its resulting effect on the approach behavior of moth prey. At base, the structure of the environment influences a species’ exposure to ambient conditions, such as temperature and airflow, and influences the distribution of species, both predator and prey. Relying on pheromones, bolas spiders are especially susceptible to changes in prey distribution and wind. The habitat and use of a bolas by the previously studied species *Mastophora hutchinsoni* both serve as a great minimalist comparison to *C. akermani*. For both species, the key first step in prey capture is attracting the moth to themselves.

A tree seems to be a simpler hunting environment for bolas spiders than an open field. This statement is based on two factors (1) natural airflow and (2) pheromone interaction with other bolas spiders. When *M. hutchinsoni* sits within a tree, its pheromones are contained within the canopy of the tree, except for the air which billows out from under the tree leaves (Figure 6). Our models predict that spinning within a tree does little to affect the natural airflow, as the tree structure directs airflow, making spider movement redundant. The presence of multiple spiders seems advantageous in this way, turning the tree into a pheromone beacon to the communal hunting ground. Once the moths are within the tree, they can follow the natural pheromone gradients to find the closest spider. They then hover close to the spider, sometimes even touching it [18,33,34]. Thus, it makes sense this species creates significantly shorter bolas, and with only a single glue droplet [5,15,16]. Only when the moth is near it, does the spider flick its bolas. This simplicity in hunting is a consequence of the moths’ ease at finding the spider.

For *C. akermani*, its hunting habitat is much more complicated as it sits in an open field, especially susceptible to wind. Persistent and strong winds, blow its small and slowly diffusing pheromone cloud in multiple directions, thinning it out and complicating the following of its gradient. Changes in airflow direction and intensity can lead to varying and cluttered gradients, limiting the ability of the moth to locate the spider. This confusion could be especially apparent when multiple spiders are positioned close to one another. Wind can mix the pheromone trails of spiders too close to one another, perhaps sending prey in the wrong direction or towards another spider increasing the potential of intraspecies competition. From our models, the spinning of the spider fights these issues with the wind. As the spider spins, it creates flow in all directions surrounding itself. When in the direction of the wind, it helps spread pheromones forward but when fighting against the direct flow of wind pheromones are sent upward and downward while slowing down airflow in the immediate area (Figure 6). Resistance to flow from the spinning may keep pheromones from simply blowing away and making it easier to target for the moth. In this environment the spinning is doing double duty, spinning the bolas and controlling pheromone distribution.

The bolas created by *C. akermani* are three to five times longer than that created by *M. hutchinsoni* which may be a response to this inability of the moths to directly locate the spider. These bolas are generally made with two glue droplets but can sometimes be made of one or three [20,21,27]. The small distance of 2 cm between the droplets creates a larger effective zone of adhesion. Any contact with the space between the glue droplet will lead to the collapsing of the bolas around the prey, ensnaring it in both glue droplets. The spinning of the bolas takes these two linear cms of glue and rotates it about an axis. The spider spins the glue droplets twice a second and creates a larger apparent cone of adhesion. Bouncing allows the adhesive zone to spin faster and overall wider area. Thus, unfortunately, there is little observational evidence, aside from our own, to determine if captured moths are typically hovering near the spider, such as those caught by *Mastophora*, or are more aggressively flying—necessitating a rapidly moving bolas. A second advantageous consequence in the structure of the multi-droplet bolas, but potentially accidental, is the wobble and bending around the D_1_ glue droplet. The glue droplets are constructed of viscoelastic glue and extra silk thread, filling a liquid droplet [35,36,37]. The construction of the bolas using two separate threads, creates a system of two tensions, adhered by a liquid droplet acting as a joint. The bending of the droplets leads to wobbling and when spun quickly can help to further increase the effective area of the adhesive strike zone.

Here, we attempt to correlate differences in bolas construction and prey capture behavior between *C. akermani* and *M. hutchinsoni* with differences in the hunting environment. We believe that alterations such as elongation of the bolas, adding additional droplets, and spinning are advantageous when living in a large open grassland where wind can readily but randomly disperse pheromones. Such adaptations are not necessary for species such as *M. hutchinsoni* which live more enclosed environments where moths may already gather, such as a tree. The alterations to the bolas of *C. akermani* are closely tied to the behavior of its prey and future studies should aim to record the behavior of a moth being captured, as this will inform our understanding of why bolases are spun. As of now, we cannot rule out several alternative hypotheses for adaptive advantages of spinning, such as the spinning behavior being tied to a unique size or physical property of its target prey species. It also will let us confirm our hypothesis that the rotation allows the glue droplets to roll around their prey when struck, wrapping them. Observing these species with multiple cameras would also allow us to calculate the forces placed on the silk and calculate the associated material properties. We would also like to collect and test these bolas for biomechanical and chemical analysis to compare the structure and diversity of the glues within the moth-specialist subfamily Cyrtarachninae. The material diversity within the family is shown by the glue droplets of *C. akermani* which are capable of remaining as distinct droplets on a single strand without flowing with gravity into one another. Similar to *M. hutchinsoni* these glues appear thicker than others such as *Cyrtarachne akirai*’s low viscosity glue which readily flows [4,18,38]. Understanding the diversity and function of these glues has much to teach us about natural bioadhesives and has application to our own synthetic adhesives.

## Data Availability

The excel data sheet used to create Figure 3 can be downloaded as Supplementary Material.

## Supplementary Materials

The following supporting information can be downloaded at: https://www.mdpi.com/article/10.3390/insects13121118/s1. Video S1: leg waving; Video S2: creating a single bolas; Video S3: creating a double bolas; Video S4: swinging a single bolas; Video S5: swinging a double bolas. Supplementary Data File S1: The excel data sheet used to create Figure 3 can be downloaded as Supplementary Material. Supplementary Data File S2: Fusion CFD files can be downloaded for the swing + bounce condition for the field and tree.
